# Alteration of the health effects of bioaerosols by chemical modification in the atmosphere: A review

**DOI:** 10.1016/j.fmre.2023.10.017

**Published:** 2023-12-25

**Authors:** Ailin Li, Xinghua Qiu, Xing Jiang, Xiaodi Shi, Jinming Liu, Zhen Cheng, Qianqian Chai, Tong Zhu

**Affiliations:** State Key Joint Laboratory for Environmental Simulation and Pollution Control, College of Environmental Sciences and Engineering, Peking University, Beijing 100871, China

**Keywords:** Bioaerosols, Allergenicity, Chemical modification, Air pollution, Climate change

## Abstract

Bioaerosols are a subset of important airborne particulates that present a substantial human health hazard due to their allergenicity and infectivity. Chemical reactions in atmospheric processes can significantly influence the health hazard presented by bioaerosols; however, few studies have summarized such alterations to bioaerosols and the mechanisms involved. In this paper, we systematically review the chemical modifications of bioaerosols and the impact on their health effects, mainly focusing on the exacerbation of allergic diseases such as asthma, rhinitis, and bronchitis. Oxidation, nitration, and oligomerization induced by hydroxyl radicals, ozone, and nitrogen dioxide are the major chemical modifications affecting bioaerosols, all of which can aggravate allergenicity mainly through immunoglobulin E pathways. Such processes can even interact with climate change including the greenhouse effect, suggesting the importance of bioaerosols in the future implementation of carbon neutralization strategies. In summary, the chemical modification of bioaerosols and the subsequent impact on health hazards indicate that the combined management of both chemical and biological components is required to mitigate the health hazards of particulate air pollution.

## 1 Introduction

Bioaerosols are airborne biologically derived particulates that contain living or dead organisms, fragments, units, and biomolecules. Specifically, they consist of viruses, bacteria, fungal spores, archaea, algae, pollens, or other fragments and excretions that are emitted from the biosphere (e.g., soil, animals, vegetations, hydrosphere, and human activities), as well as biologically-derived molecules such as proteins and lipids [1,2]. These components are present alone or are mixed with chemical components, contributing to as much as 30% of the aerosol mass [1,^3^,4]. Like chemical aerosols, the size of bioaerosols ranges from a few nanometers to tens of micrometers. Large particles can be rapidly removed from the atmosphere by precipitation, while small particles remain suspended in the air for a long time, and can be inhaled into and deposited in the human respiratory system [5]. Normally, biological particles larger than 0.5 µm are mainly deposited in the upper airway, while smaller particles can diffuse into the deeper respiratory tract and even deposit in the lungs [6]. The health hazard of bioaerosols is one of the issues that deserve major attention.

Most studies have focused on the primary bioaerosols (PBA) emitted directly from various sources. After being emitted into the air, PBA can be modified through aging processes to generate secondary bioaerosols. Given that such modifications could substantially change their composition and further alter their health effects [1,7], determining the impact of atmospheric processes on bioaerosol components is critical to understand their health hazards.

Infectivity and allergenicity are the most typical and well-acknowledged adverse effects of inhaled bioaerosols such as pollen proteins, dust mites, bacteria, and fungal spores, which are all well-recognized allergens [8]. For example, exposure to pollen proteins and dust mites can trigger the proliferation of T cells, causing the production of immunoglobulin (Ig) and inducing allergic diseases such as asthma and rhinitis. In addition, endotoxin and glucose in the cell membrane of bacteria and fungi have been proven to bind with receptors on the surface of immune cells, thereby stimulating a series of allergic and inflammatory symptoms like fever and cough [9], [10], [11]. In a word, exposure to bioaerosols can induce negative allergic effects on humans that deserve further concern.

**Fig. 1:**
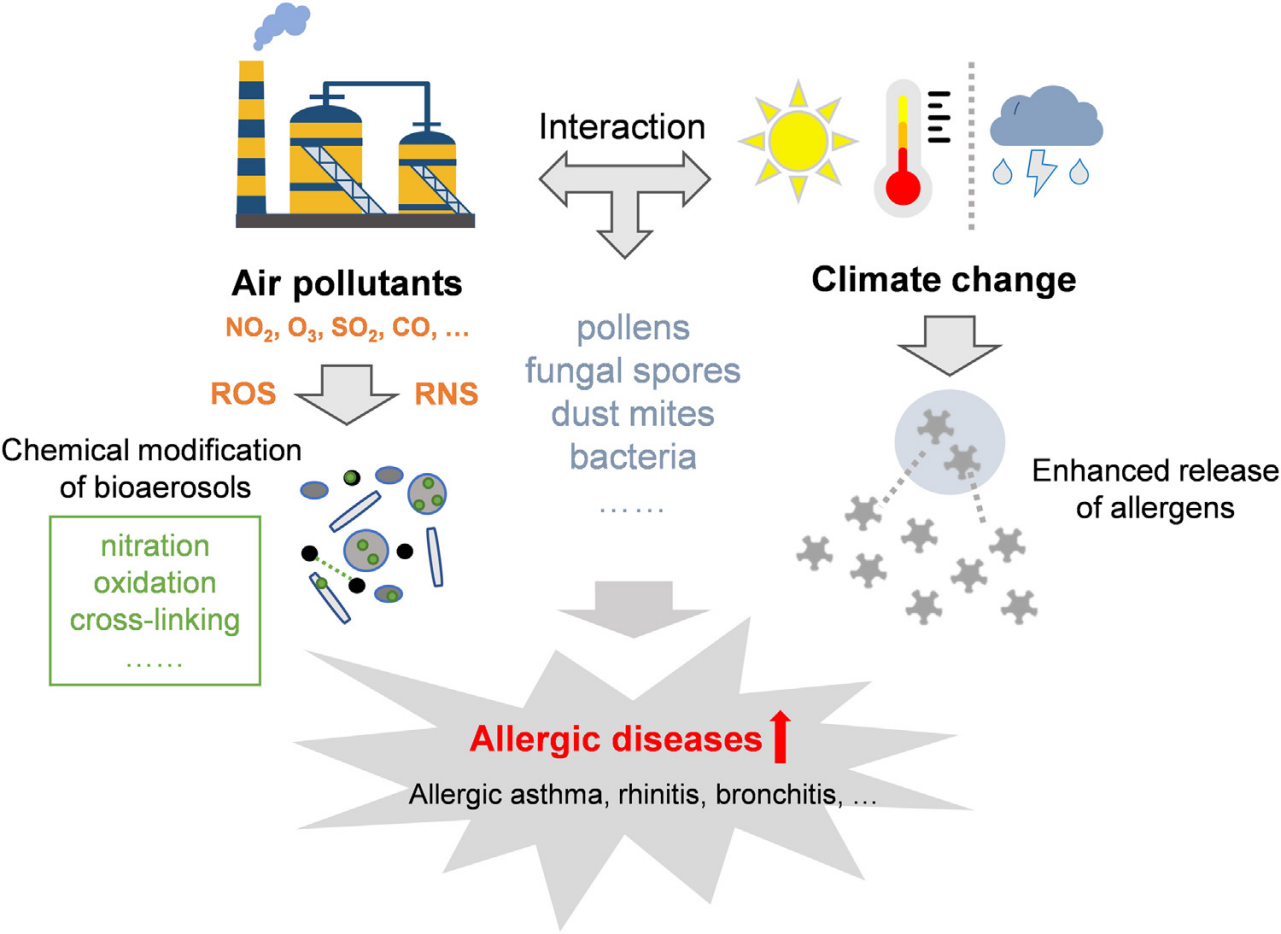
**Enhanced health effects caused by the modification of air pollutants and climate change**.

In recent decades, both the degree of susceptibility to specific allergens and the prevalence of allergic diseases have increased worldwide [12,13]. The cause is not fully understood; however, many studies have attributed this to the increasing levels of air pollution, especially in urbanized societies with intensive anthropogenic emissions such as nitrogen oxides (NO_x_), carbon dioxide (CO_2_), ozone (O_3_), carbon monoxide (CO), sulfur dioxide (SO_2_), and volatile organic compounds [14], [15], [16], [17]. Air pollutants can increase the reactivity, i.e., oxidation capacity, of the atmosphere, in turn promoting the transformation and functionalization of particulate chemical components. Similarly, bioaerosols are also likely altered under such conditions, consequently leading to the alteration of their health effects, especially allergic sensitization (Fig. 1). Such modifications usually occur on the biomolecules of proteins, lipids, and polysaccharides, which further alter their structures, functions, and toxic properties. However, few studies have summarized the aging processes and their impacts on the health effects of bioaerosols, which is a key issue in the context of the interactions between air pollution and climate change.

To provide a systematic understanding of the adverse effects of bioaerosols, in this review we summarized the chemical modification of bioaerosols in the atmosphere, with a focus on the alteration of allergenicity after the aging process. Furthermore, we proposed the mechanisms by which allergenicity is enhanced and considered the interactions among bioaerosols, air pollutants, and climate change. As a result, we were able to recommend the critical issues that should be investigated in the future.

## 2 Atmospheric chemistry of bioaerosol components

Chemical reactions are key atmospheric processes, which can facilitate the transformation and removal of air pollutants. These processes can also occur on PBA. After emission, PBA components, i.e., amino acids, peptides, proteins, lipids, sugars, and other biomolecules, can be chemically modified by NO_2_, O_3_, radicals, and other trace gases, resulting in the generation of secondary bioaerosols. For example, reactive oxygen/nitrogen species (ROS/RNS) in the atmosphere can induce heterogeneous or multiple-phase chemical reactions, leading to the oxidation, nitration, oligomerization, or degradation of peptides or proteins; this results in an enhancement of the allergenicity of pollens, bacteria, and fungal spores [12,[18], [19], [20]]. Several major chemical processes including oxidation, nitration, oligomerization, and other reactions are described further in Sections 2.1 to 2.3.

### 2.1 Oxidation by hydroxyl radicals and ozone

Hydroxyl radicals (OH·) are generated by complex photochemical reactions and are the most reactive ROS in the daytime. They can react with biomolecules through addition to the double bond or abstraction of the hydrogen atom in unsaturated and saturated biomolecules, respectively, and then initiate further oxidation reactions. OH· can also oxidize the sulfhydryl group of cysteine in glutathione (GSH) to sulfenic acid (GSOH), sulfinic acid (GSO_2_H), and sulfonic acid (GSO_3_H) [18,[21], [22], [23], [24]]. Moreover, a series studies have proven that OH· and other ROS could be absorbed onto the surface of bioparticles or penetrate into bacterial cell to oxidize amino acids, proteins, fatty acids, and even the whole organisms, resulting in an irreversible inactivation [25,26]. For instance, study has demonstrated that bacterial cells could be inactivated and decomposed by the attack of photogenerated H_2_O_2_ from OH· , and the damage of cell membrane and bacterial proteins was also observed [27]. Other ROS such as O· and halogen atoms (chlorine, bromine, and iodine) can also oxidize bacteria, viruses, fungi, pollens, and other organisms, resulting in their eventual damage and inactivation [25,[28], [29], [30]], and oxidative damage can also be induced by some photocatalytic process of biological compounds [31,32].

**Fig. 2:**
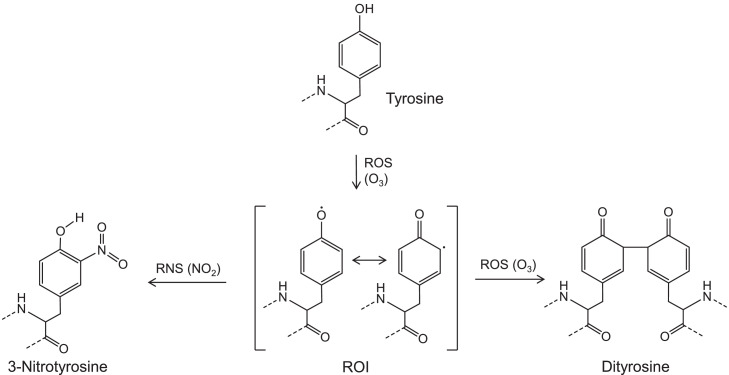
**The nitration and oligomerization mechanisms for tyrosine in airborne bioaerosols**[20].

Similar to OH·, O_3_ can trigger the oxidative modification of proteins and initiate further nitration or oligomerization. For instance, samples collected from industrially contaminated sites showed significant oxidation modification of amino acids such as methionine, histidine, lysine, and proline. This result has been hypothesized to be related to the enhancement of oxidation caused by the higher O_3_ concentrations found in polluted regions than in clean areas [33]. Additionally, after O_3_ exposure, the absorption and emission spectra of aromatic amino acids were found to change, indicating their transformation. Currently known reactions of aromatic amino acids after ozone exposure are oxidation and hydrolysis of tryptophan to produce kynurenine, and oxidation of tyrosine to produce phenoxy reactive oxygen intermediates (ROI) followed by nitration or oligomerization process [28,34]. As shown in Fig. 2, the ROIs generated via tyrosine oxidation can be nitrated in the presence of NO_2_. On the other hand, ROIs can also undergo a cross-linking reaction to produce dimers, trimers, and higher oligomers after constant exposure to O_3_ without NO_2_. Both nitration and oligomerization can change the structures of proteins and therefore affect the functions including health effects on humans, especially on the respiratory and immune systems [17,^18^,^20^,35]. However, the mechanism is not fully understood regarding the competition between oxidation and oligomerization, and the reactions with tryptophan and phenylalanine have been less well-studied than those with tyrosine. In addition to oxidation and cross-linking, trace concentration of O_3_ have also been proved to cause inactivation of bioaerosols such as bacteria, which can be applied to prevent the spread of microbial pathogens in the air [36].

### 2.2 Nitration by reactive nitrogen species

After activation by OH· or O_3_, bioaerosols can be further nitrated in the presence of RNS, which consist of NO_2_, NO_2_·, NO_2_^+^, NO_3_·, and N_2_O_5_. The susceptibility of the resonant structures of phenoxyl radical to nitration makes tyrosine residues (containing both an aromatic ring and phenolic OH) of proteins a classic substrate for reactions [19,[37], [38], [39]].

As shown in Fig. 2, tyrosine can be stimulated by O_3_ into ROIs, after which the tyrosyl radical can form 3-nitrotyrosine via electrophilic substitution by NO_2_ [20]. A simulation study observed such a mechanism in actual pollen proteins. Franze et al. illustrated that *Betulaceae* pollen proteins with the allergen Bet v 1 (a molecular weight of 17 kDa and seven tyrosine residues) could be efficiently nitrated by traffic-related pollutants (NO_2_ + O_3_), and the nitration degree of proteins increased continuously with the elevation of both the level of nitration agent and exposure time [12]. Nevertheless, the nitration degree reduced substantially without the presence of O_3_ or sufficient relative humidity (RH), suggesting the importance of ROS and the hydration state for the nitration process, as shown in Eq. 1:

(1) $$\text{ROS}+NO_{2}+H_{2}O\to \;\cdot NO_{3}/N_{2}O_{5}/\text{HN}O_{3}$$

Except for the heterogeneous nitration reaction induced by NO_2_ and O_3_, the nitrate ion (NO_3_^−^) can also serve as an RNS and induce the nitration of pollen allergens in the simulated liquid phase of particles [40]. Given the large proportion of nitrate in particulate matter, Ghiani et al. reported for the first time that proteins in *Phleum pratense* pollens (allergen Phl p 2) could be nitrated in the aqueous phase under an appropriate NO_3_^−^ concentration, pH, and ultraviolet (UV) irradiation. Under such photochemical conditions, NO_3_^−^ can be transformed into NO_2_· and OH· (Eq. 2), resulting in the formation of 3-nitrotyrosine.

(2) $${\text{NO}}_{3}^{-}+h\nu +H^{+}\to \;\cdot NO_{2}+\cdot \text{OH}$$

Briefly, nitration is an important atmospheric aging process for bioaerosols along with oxidation. Bioaerosols can be chemically modified during this process, leading to an enhancement of their allergic effects after deposition in the respiratory system.

### 2.3 Other reaction processes

Air pollutants such as SO_2_ can also impact the composition and sensitization of biological components. Anthropogenic pollutants may affect the surface or inside of biological particles, and even generate new species [41,42]. Studies have found that SO_2_ can heterogenous uptake onto the oleic acid and other unsaturated fatty acid, and further reacts on the surface or within the particle phase, leading to the generation of organosulfates. Such reactions are important to the study of secondary organosulfur compounds, and their adverse health effects deserve further attention [43], [44], [45]. Additionally, it has been speculated that nitrated-proteins can be involved in photochemical reactions. For instance, 3-nitrotyrosine was found to degrade during long-term solar radiation exposure, leading to the release of nitrous acid (HONO) from intramolecular H transfer. However, contribution of such reaction to the atmospheric oxidation capacity remains unclear [46].

In addition to proteins, lipopolysaccharide (LPS), a type of bacteria-originated endotoxin, can undergo chemical transformations. LPS is a major biomolecule in the outer membrane of gram-negative bacteria, and is composed mainly of polysaccharide and lipid A. As a ligand molecule, LPS can bind to antibodies on the surface of human immune cells and trigger an inflammatory response. Previous studies reported that LPS on the surface of sea spray aerosols could heterogeneously react with HNO_3_, contributing to the atmospheric nitrate salts (as shown in Eq. 3, 4), and the reactive sites were proved to be the carboxylate and phosphate groups of LPS [18,47]. This finding revealed a new pathway for the formation of nitrate, and the alteration of toxicity during such processes requires further attention.

(3) $$\text{HN}O_{3\left(g\right)}+R-\text{COON}a_{\left(p\right)}\to \text{NaN}O_{3}+R-\text{COOH}$$

(4) $$\text{HN}O_{3\left(g\right)}+R-\text{HP}O_{4}Na_{\left(p\right)}\to \text{NaN}O_{3}+R-H_{2}PO_{4}$$

In addition, solar radiation and high temperature can promote the aging of bioaerosols. Under UV irradiation or in a high-temperature climate, bacteria, viruses, spores, and other biological compounds can be inactivated, causing DNA damage or the apoptosis of cells in bioaerosols. Both high temperature and irradiation are common techniques for the purification of infectious aerosol pollution in the air. RH can also affect bioaerosol activity to an extent. For example, with a decrease in RH, the viability of bacteria generally decreases. However, for viruses, both low and high RH are conducive to their generation, whereas a moderate RH may lead to their inactivation [18,28].

## 3 The alteration of the health effects of bioaerosols

Bioaerosols can cause a series of adverse health effects including infection, allergy, and respiratory inflammation. Allergies and related diseases are a major concern, although the inducement of these conditions by bioaerosols is not fully understood. In addition to genetic factors, allergic diseases were partly attributed to anthropogenic air pollution (i.e., SO_2_ and fine dust from fossil fuel combustion) in early studies. However, recent studies have shown that increasing levels of traffic-related pollutant of NO_2_ and photochemical pollutant of O_3_ in urban areas could provide a molecular rationale for the exacerbation of asthma, rhinitis, and other allergic symptoms [48]. In addition to the direct inhalation of these air pollutants, some results have indicated that bioaerosol components, such as proteins, might be chemically modified by ROS and RNS (as shown in Section 2), altering their structure and further enhancing allergenicity [49], [50], [51]. In a word, in an oxidizing environment, the impact of atmospheric chemical modification is a key issue on the health effects of bioaerosols.

### 3.1 Impact of nitration

The nitration process occurs in both the atmosphere and the human body. In the physiological processes in the human body, nitrated proteins are regarded as a hallmark of inflammation. While in the atmosphere, nitration is usually a post-translational modification (PTM) of biological compounds. The main RNS involved in the nitration of bioaerosols are NO_2_, NO_2_·, and NO_3_^−^. Tyrosine, one of the most effective amino acids in mediating molecular recognition, is a well-recognized target of nitration. Several studies have demonstrated that such PTMs might generate new antigenic epitopes and induce the T-cell response. Compared with unmodified bioaerosols, modified bioaerosols have a higher potential to trigger immune responses and allergenicity [52,53]. The modification targets of nitration mainly include pollen proteins, fungal spores, and indoor dust mites.

#### 3.1.1 Pollen proteins

Pollens are important carriers of allergen proteins and can cause allergic diseases after inhalation. Although pollen concentrations are typically lower in urban than rural areas, urban populations have a higher percentage of allergy sufferers [54]; this is suggestive of the modification of proteins by urban air pollutants. It has been reported that the most abundant allergen, Bet v 1 of birch pollen, is more liable to be nitrated than other proteins, and the preferred reaction sites by NO_2_ and O_3_ with tyrosine residues might be Y83 and Y158 [53]. The nitrated Bet v 1 had a significantly enhanced binding capacity with immunoglobulin E (IgE), where a higher degree of nitration yields stronger allergenicity [12,[55], [56], [57]]. Similarly, the sensitization potential of allergen Phl p 5 from timothy grass pollen, which includes 12 tyrosine residues and accounts for 6% of the whole pollen extract, could also be increased under the modification of NO_2_ and O_3_ [38,^58^,59]. The *Platanus* pollen allergen a 3 (Pla a 3) also exhibited enhanced allergenicity after exposure to NO_2_ and O_3_, and an in vivo experiment proved that the nitration process could greatly aggravate pollen-induced pneumonia [60].

In addition to nitration by NO_2_ and O_3_, exposure to NO_2_ alone can also enhance pollen-driven respiratory allergies. For instance, NO_2_ exposure may increase the germination rate of pollen, which might enhance its maturity and diffusion, which are two key processes for inducing allergies. Moreover, NO_2_ can cause oxidative stress and increase superoxide dismutase (SOD) activity in pollen cells, thereby promoting the combination of allergens and antibodies [61]. In addition to nitration, NO_2_ could induce the nitrosylation of pollen proteins. For example, the sulfhydryl group (-SH) of cysteine can be nitrosylated into -SHNO, and enhanced NO_2_ exposure is associated with a higher allergy risk [62].

Interestingly, the effects of NO_2_ and O_3_ on allergenicity are sometimes antagonistic. A laboratory simulation study showed that the allergenicity of nitrated Pla a 1 and Pla a 2 induced by NO_2_ and O_3_ was lower than that by NO_2_ exposure alone [63]. In field observations, the allergenicity of ragweed pollen collected near a highway had a significantly positive correlation with the NO_2_ concentration, but not with the O_3_ concentration [64]. Although further evidence is required to confirm these results, some studies have suspected that O_3_ might cause the denaturation of proteins, altering their structure and function. For instance, O_3_ might contribute to the formation of disulfide in cysteine-containing peptide chains, interfering its further reaction with NO_2_ and possibly inhibiting the nitration process [65,66].

#### 3.1.2 Fungal spores

Exposure to fungal spores of *Aspergillus fumigatus* can lead to allergic bronchopulmonary aspergillosis, cystic fibrosis, chronic asthma, and other allergic diseases [67]. Lang-Yona et al. [16] explored the allergenic activity of the asexual conidia of *A. fumigatus* under ambient and laboratory conditions. Compared to the unexposed control group, the allergenicity of modified spores increased more than 2-fold during an exposure time of 0–12 h, presumably from the contribution of protein nitration. However, the allergic potency decreased with the extended exposure time, possibly due to protein deamidation. These results indicated that the promotion of allergenicity by nitration might have a threshold of exposure time, with a long exposure resulting in excessive modification, i.e., protein deamidation, which might mitigate the effect of nitration.

#### 3.1.3 Indoor dust mites

Because humans spend most of their time indoors, the biological components of house dust, especially house dust mites (HDMs), are one of the most important allergens. In addition to inhalation, HDMs can cause a type I immune response through skin contact [68]. *Dermatophagoides farina* (Der f 1) and *Dermatophagoides pteronyssinus* (Der p 1) are the major HDM allergens that can bind to IgE, and are responsible for the allergic effects in > 50% of patients [69]. Recently, Xu et al. [70] measured the concentration of Der f 1 and Der p 1 and their nitrated products in dormitory dusts, and showed that the accumulated levels of nitrated HDMs were higher than those of non-nitrated HDMs. Moreover, the risk indexes were high for nitrated HDMs, suggesting the importance of nitration on allergens.

### 3.2 Impact of oxidation

The oxidative modification of bioaerosols is believed to be related to air pollutants, which can enhance the allergenicity of proteins. After exposure to O_3_ and NO_2_, proteins such as Pla a 3 can be oxidized. According to liquid chromatography-tandem mass spectrometry measurements, in addition to the nitration of tyrosine, the methionine at sites 1 and 17 of the Pla protein was oxidized; this might contribute to the stability of allergens [60]. Smiljanic et al. [33] compared pollen samples collected in polluted and clean environments, and observed 2.3–14.7 times more oxidative modification in polluted samples compared to clean samples, especially on the histidine residues in polluted samples. Additionally, O_3_ could modify the lipids of cytoderm, influencing the immune adjustment. It could also react with the components in the pollen coating to produce 4-hydroxybenzaldehyde, which might impact the germination and allergenicity of ambient pollens [71].

In summary, oxidative modification could partly explain the increased allergenicity of bioaerosols. Given that oxidation is typically accompanied by nitration, the degree of their contributions and potential competition between these reactions to the enhancement of allergenicity require further investigation.

### 3.3 Impact of cross-linking

Like nitration and oxidation, oligomerization via the cross-linking of proteins in bioparticles can result in irreversible structural and functional changes. Taking tyrosine as an example, a flow tube study observed the generation of Tyr-Tyr-dimers and higher oligomers upon O_3_ exposure under ambient conditions [35]. The chemical bond between tyrosine was considered to be an intermolecular covalent bond. The study also showed that with exposure to O_3_ and NO_2_ at environmentally relevant levels, up to 50% of Phl p 5 proteins were converted into oligomers through cross-linking, together with 10% of nitrated tyrosine residues. These results indicate that nitration and cross-linking of bioaerosols can occur simultaneously, and they may be competitive processes. These two modifications could lead to the alteration of the immunogenicity and allergenicity of bioaerosols by affecting signal transduction among pro-inflammatory cells [38]. However, their relative contribution to the enhancement of health effects remains unclear. The cross-linking of cysteine was also found to affect allergenicity in the presence of O_3_ and NO_2_. An in vivo study suggested that under the conditions of O_3_ and NO_2_ pollution or with O_3_ alone, a new disulfide bond could be formed between two sulfhydryl groups of adjacent cysteine residues in the peptide chain, which had a similar impact on the health effects of bioaerosols as the oligomerization of tyrosine [72,73].

Currently, little is known about the oligomerization of amino acids other than tyrosine and cysteine in the presence of O_3_ and NO_2_; therefore, further investigation is required.

### 3.4 Impact of other factors in real environment

Real atmospheric environments are complex, and host air pollutants other than NO_2_ and O_3_ may impact the health effects of bioaerosols. For example, SO_2_ levels have been reported to be related to the alteration of allergenicity, inflammation, and viral infectivity of bioaerosols [41,42]. Unlike RNS, which enhance allergic effects via nitration, SO_2_ cannot induce nitration. Whether SO_2_ induces chemical modification of proteins in bioaerosols remains unclear, and the mechanism controlling its effect on allergic potential merits further exploration.

In addition to allergic diseases, exposure to air pollutants is associated with an observed increase in respiratory viral infections. For example, NO_2_, SO_2_, CO, and other pollutants might aggravate some infectious diseases such as influenza, mumps, and viral pneumonia. Exposure to air pollutants may affect the life cycle of viruses, promote viral replication, and promote their ability to penetrate human epithelial tissues, thereby facilitating spread and enhancing inflammation and damage upon infection [74]. Moreover, short-term exposure to particulate matter has been proved to associated with the increase of lower respiratory infection; however, in addition to the individual effect of particulate matter, the interactions between chemical and microbiological components may also play an important role [75].

Different from simulation studies typically on single pollutant, the real atmosphere contains countless compositions of pollutants under complex conditions. Pollution levels might greatly affect the diversity and composition of bioaerosols [76,77]. Taken haze events as an example, high relative humidity, strong oxidation capacity, interaction between various gaseous pollutants, diverse particle components, anthropogenic activities, and other factors jointly result in haze events with complicated conditions, which might have combined effects on the load and components of bioaerosols and further change their health effects [78]. An observational study in Xi'an showed that the total airborne microbe concentration in bioaerosols on haze days was higher than that on non-haze days, and the diurnal variation peak was consistent with the traffic peak. These results indicated that haze may lead to an increased risk of infectious pneumonia and even allergic effects, and the atmospheric oxidation capacity would likely have a significant impact on bacterial communities during haze events [79]. For some polluted areas such as landfill, the diverse pollutants and complex microbial communities may accelerate the development of pathogenic bacteria and antibiotic-resistance genes. The long-term inhalation of such bioaerosols in the surrounding area has been considered to have a nonnegligible health risk especially for the vulnerable individuals [80]. Therefore, in addition to laboratory simulations, field studies are warranted to evaluate the health effects of bioaerosols in the real atmosphere, which is of great significance for the controlling of such pollutants and for the protection of public health.

## 4 The mechanism of toxicity alteration induced by the modifi-cation of bioaerosols

**Fig. 3:**
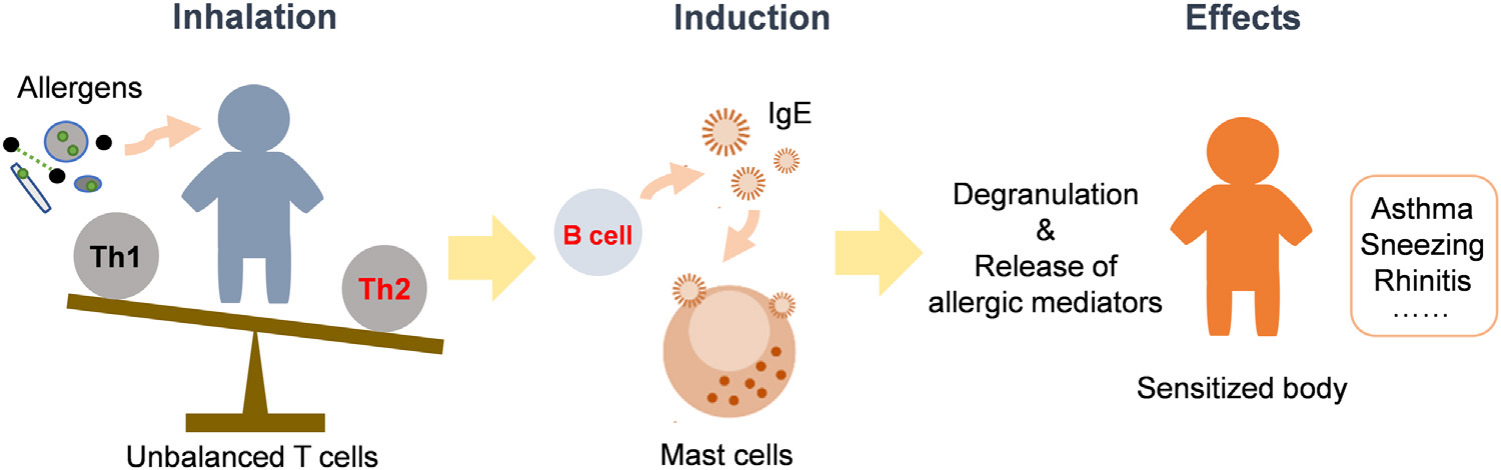
**Pathways of allergenicity induced by allergen exposure**.

The enhancement of allergenicity is one of the most typical toxicity alterations of modified bioaerosols. Allergic diseases, including asthma, rhinitis, bronchitis, etc., are common type I-mediated allergies caused by bioaerosols such as pollens, dust mites, and fungal spores [59]. Type I allergies are generally mediated by IgE antibodies. After exposure to the allergen, T cells proliferate and induce B cells to secrete IgE antibodies, which can be adsorbed on mast cells or basophils and cause the body to enter a sensitized state. Upon re-exposure to the allergen, the sensitized protein of the allergen can bind to IgE and cause further allergic reactions (Fig. 3).

Normally, T helper 1 and 2 (Th1 and Th2), which are two subsets of T cells, are in equilibrium in the human body. Th2 cells can induce anaphylaxis and assist B cell activation to promote the generation of IgE antibodies. The imbalance of Th1 and Th2 cells caused by nitrated bioaerosols could be an important mechanism by which the allergic effects of bioaerosols are enhanced. Studies have shown that nitrated Bet v 1 could inhibit the production of cytokines that initiate the Th1 allergic responses, thereby shifting the balance toward Th2 and enhancing the allergenicity [51]. Compared with non-nitrated Bet v 1, when mice were exposed to nitrated Bet v 1 allergen, the proliferation of splenocytes and secretion of the inflammatory factor interleukin (IL)−5 were significantly increased. The IgE and IgG levels in serum also increased, indicating that the nitrated allergens could promote the secretion of more Ig. In addition, immunoblot and enzyme-linked immunosorbent assay results revealed a significant increase in the combination of nitrated allergens with IgE antibody in the serum of patients, and the nitration process enhanced the cross-reaction with other allergens. These findings indicate that exposure to nitrated proteins might enhance the IgE-mediated immune response, and a mono-sensitive patient might be converted into a poly-sensitive patient, thereby increasing the risk of allergic disease [40,^42^,81].

Exposure to nitrated bioaerosols may also enhance oxidative stress, causing the aggravation of allergen-induced diseases such as pneumonia. In a recent study [60], an in vivo experiment on a mouse model showed an increased number of inflammatory cells in pathological sections of lung tissues after exposure to bioaerosols together with NO_2_, O_3_, and their mixtures when compared with mice exposed to non-nitrated allergens. Malondialdehyde and SOD levels in lung tissue were higher in exposed mice than control mice. In addition, the combined exposure of NO_2_ and O_3_ to Pla a 3 resulted in further increases in interferon-γ and IL-4 levels in the sensitized mice.

Furthermore, the impact of the chemical transformation of bioaerosols on allergic effects can be explained in terms of alterations to the protein structure and disruption to the integrity of the cell membrane. Oligomers generated through oxidative cross-linking provide more epitopes on the protein surface and promote their binding to IgE antibodies. In addition, cross-linking can reduce the hydrolysis of allergen proteins to enhance their stability [17]. Smiljanic et al. [33] used electron microscopy to compare the structure of pollen samples from regions with intensive traffic flows and industrial pollution with those from a conservation area. They found that pollens from environmental protection areas had a more regular structure, whereas pollens from polluted regions had thinner outer walls and irregular long spines. Spectroscopic analysis (i.e., infrared spectroscopy and X-ray photoelectron spectroscopy) also revealed that the spectral characteristics of the pollen proteins changed after exposure to NO_2_ and O_3_, e.g., the protein conformation of the pollen wall had been altered [63]. The structural characterization of Pla a 3 before and after exposure to NO_2_ and O_3_ revealed that proteins in the unexposed group were more susceptible to degradation, and the permeability test of the cell membrane by Trypan blue staining also suggested damage to pollen cell membranes after a combined exposure to NO_2_ and O_3_, which could increase the release of allergic components in pollen to an extent [60]. These results suggest that allergenic bioparticles could undergo further chemical, physical, and biological modifications when exposed to atmospheric pollutants, which could damage their structure and affect their function and health effects.

## 5 The interactions among climate change, pollutants, and bio-aerosols

In addition to the chemical modifications of biological components by air pollutants, extreme climate events and global warming can affect the concentration and distribution of bioaerosols. The interactions among climate change, pollutants, and bioaerosols could aggravate the adverse effects of bioaerosols on human health.

The atmospheric CO_2_ concentration is the main factor controlling the global greenhouse effect. Elevated CO_2_ concentrations lead to global warming and affect environmental acidity, which have been proposed by some studies to alter the physiological properties of the biological components of atmospheric aerosols, such as fungal spores, and further affect their health effects, especially respiratory sensitization [82,83]. Lang-Yona et al. [15] showed that the allergenicity of *A. fumigatus* increased by up to 8.5 fold at the current levels of atmospheric CO_2_ compared with the pre-industrial age. An increase in the atmospheric CO_2_ concentration might affect the respiration rate of fungus and the carbon/nitrogen content of the growth medium, which might then contribute to the enhanced allergenic effect of the fungus. Additionally, changes in air temperature and extreme weather could affect pollen and spore populations. Higher temperatures and higher pollen concentrations in summer often amplify the rate of allergy sufferers [13]. The occurrence of extreme weather such as thunderstorms has also been found to be associated with the prevalence of asthma, especially during the pollen transmission season. During thunderstorms, pollen is carried to the base of clouds, where it breaks up and is transported to the ground via fragments, causing a rapid increase in airborne allergen concentrations before rainfall. This specific allergic phenomenon is commonly known as thunderstorm asthma [84]. In addition to the direct effect on bioaerosols, climate change could also indirectly exacerbate the health hazards of bioaerosols by promoting air pollution, although little attention has been given to this issue. For example, extremely dry weather can cause pollution events such as sandstorms, but whether such an interaction will lead to an increased risk of allergic diseases remains unclear.

There is a need for further studies on the synergistic effects of climate change, air pollution, and bioaerosols, as well as the development of measures to control pollution and health hazards. Specifically, the implementation of carbon neutrality policy, energy reform, emission reduction, and the combined management of PM_2.5_ and O_3_ would help to gradually achieve the goals of increased atmospheric visibility, reduced carbon emissions, and reduced atmospheric oxidation. Such an ameliorative atmospheric background may mitigate the enhanced health effects of both the biological and chemical components of particulate matter.

## 6 Conclusion and future perspectives

In an oxidative atmosphere, bioaerosols can be transformed via reactions with RNS and ROS, and may be impacted by climate and weather factors. A series of chemical modifications could change the structure and function of bioparticle components. Such alterations could trigger an exacerbation of adverse effects, especially allergenicity, through pathways such as enhanced affinity with IgE and oxidative stress levels. Despite the advances made in understanding these issues, many challenges remain.

First, the alteration of tyrosine has been well studied but the modification of other biological components still needs to be resolved, e.g., LPS, non-sensitizing adjuvants in pollen (enzymes and dextran), and other aromatic amino acids. Moreover, it is unclear whether RNS other than NO_2_ and NO_3_^−^, such as NO_2_·/NO_3_· and NO_2_^+^, can mediate the nitration of biological components in atmosphere.

Second, most studies have explained the alteration of the health effects of modified bioaerosols from the perspective of single-pollutant exposure, typically through laboratory simulations. However, the atmospheric environment contains complex pollutant compositions, which might lead to synergistic or antagonistic effects. Thus, the changes in bioaerosol components and their health effects in the real atmosphere need further study; however, the identification of novel biological components represents a big challenge for the lack of prerequisite knowledge. On this issue non-targeted analytical techniques based on gas or liquid chromatography and high-resolution mass spectrometry provide a promising solution, which can obtain the accurate molecular weight of specific components and therefore be helpful for the identification of such modified biological components such as nitrated proteins and oxidized lipids. Application of non-targeted technique for unknown biological components is anticipated to be an important strategy for bioaerosol studies.

Third, the current focus on the health hazards of modified bioaerosols mainly includes allergic diseases, with little concern for other health outcomes. Moreover, in addition to toxicological results, evidence from epidemiological experiments is needed to confirm and quantify the alteration of health effects from the modification of bioaerosols.

Under a context of increasing atmospheric oxidative capacity and global climate change, the epidemic of allergic diseases in recent years has highlighted the need to study the modification of bioaerosols and their altered health effects. This is essential for the formulation of effective control measures and the combined management of chemical and biological components, including the mitigation of their health hazards, especially respiratory diseases such as allergic asthma, rhinitis, bronchitis, and lung function impairment.

## Declaration of competing interest

The authors declare that they have no conflicts of interest in this work.
